# Structures of RNA Polymerase Closed and Intermediate Complexes Reveal Mechanisms of DNA Opening and Transcription Initiation

**DOI:** 10.1016/j.molcel.2017.05.010

**Published:** 2017-07-06

**Authors:** Robert Glyde, Fuzhou Ye, Vidya Chandran Darbari, Nan Zhang, Martin Buck, Xiaodong Zhang

**Affiliations:** 1 Section of Structural Biology, Department of Medicine, Imperial College London, London SW7 2AZ, UK; 2 Department of Life Sciences, Imperial College London, London SW7 2AZ, UK

**Keywords:** transcription initiation, DNA opening, transcription bubble, sigma factor, RNA polymerase, sigma54, AAA protein, transcription intermediate complex, transcription closed complex, DNA distortion

## Abstract

Gene transcription is carried out by RNA polymerases (RNAPs). For transcription to occur, the closed promoter complex (RPc), where DNA is double stranded, must isomerize into an open promoter complex (RPo), where the DNA is melted out into a transcription bubble and the single-stranded template DNA is delivered to the RNAP active site. Using a bacterial RNAP containing the alternative σ^54^ factor and cryoelectron microscopy, we determined structures of RPc and the activator-bound intermediate complex en route to RPo at 3.8 and 5.8 Å. Our structures show how RNAP-σ^54^ interacts with promoter DNA to initiate the DNA distortions required for transcription bubble formation, and how the activator interacts with RPc, leading to significant conformational changes in RNAP and σ^54^ that promote RPo formation. We propose that DNA melting is an active process initiated in RPc and that the RNAP conformations of intermediates are significantly different from that of RPc and RPo.

## Highlights

• RNA polymerase closed complex (RPc) structure reveals DNA distortions by σ
• Intermediate complex (RPi) structure reveals the roles of AAA activator
• DNA distortion and opening are initiated in RPc and RPi before entering the RNAP
• RNAP conformation in RPi is significantly different from closed or open complex

## Graphical Abstract

**Figure:**
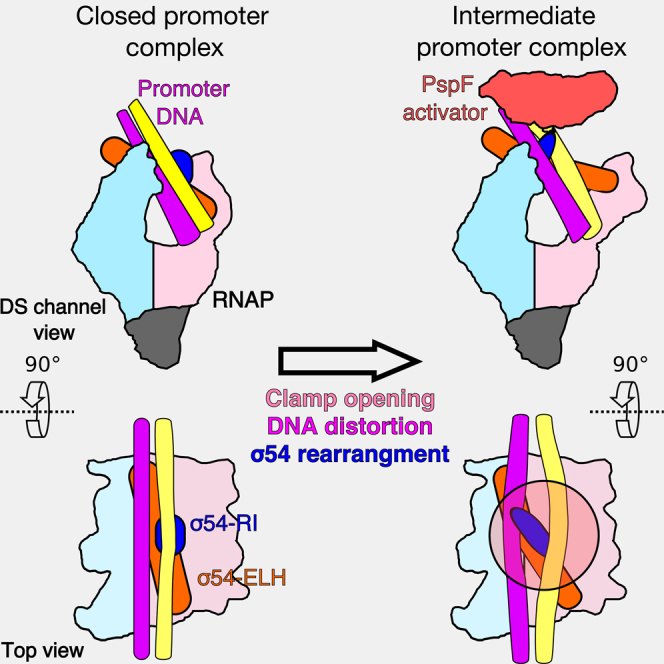

## Teaser

Glyde et al. report structures of σ^54^-dependent RNA polymerase (RNAP) closed (RPc) and intermediate (RPi) complexes. Promoter DNA distortions occur in RPc and RPi. Transitions from RPc to RPi are accompanied by significant conformational changes in RNAP and σ^54^. Direct interactions between AAA activators and DNA contribute to DNA distortions.

## Introduction

Gene transcription is a fundamental process carried out by RNA polymerase (RNAP), which is conserved from bacteria to humans. The bacterial RNAP has a core consisting of β, β′, two α, and ω subunits (Cramer, 2002, Werner and Grohmann, 2011). For transcription to occur, the closed promoter complex (RPc), where DNA is double stranded and the transcription start site (TSS; +1) DNA lies outside the RNAP, must isomerize into an open promoter complex (RPo), where the DNA is opened into a transcription bubble between −10 and +2 (relative to TSS) and the template strand is delivered to the RNAP active site (Bae et al., 2015, Murakami et al., 2002, Zhang et al., 2012b, Zuo and Steitz, 2015). The obligatory isomerization process from RPc to RPo is anticipated to involve multiple intermediate states of RNAP and DNA and is still relatively poorly understood (Gries et al., 2010, Grünberg and Hahn, 2013, Hantsche and Cramer, 2016). Multiple factors are involved in bringing RNAP to the specific promoter DNA sites where transcription is carried out (Browning and Busby, 2016, Vannini and Cramer, 2012). In bacteria, dissociable sigma (σ) factors are utilized to recruit RNAP to the promoter sites (Feklístov et al., 2014, Mooney et al., 2005). The σ factors can be broadly grouped into σ^70^ and σ^54^ classes. The σ^70^ class recognizes consensus promoter sequences at −10 and −35 (upstream relative to TSS at +1) and can form an RPo spontaneously. Many activators of σ^70^ act as recruitment factors to increase RPc formation through interactions with C-terminal domain of α subunit. σ^70^ activators can be broadly grouped depending on the binding sites of the activator on DNA: class I activators bind further upstream, while class II activators bind closer to the promoter region (Browning and Busby, 2004, Browning and Busby, 2016). The major variant sigma factor (σ^54^) forms a class on its own and controls stress-related gene expression, including heat shock, membrane stress, and nutrient starvation genes, thus playing important roles in bacterial adaptation and pathogenicity (Buck et al., 2000). σ^54^ brings RNAP to the promoter DNA through binding to consensus sequences at −12 and −24 and forms a stable closed complex that rarely isomerizes to an RPo. Instead, activator proteins belonging to the large ATPase associated with diverse cellular activities (AAA) family that bind remotely at enhancer-like sites upstream of the RPc are required (Rappas et al., 2007). The activator proteins, which are also known as bacterial enhancer-binding proteins (bEBPs) and consist essentially of an AAA domain, use their ATPase activity to remodel the RPc into the RPo in order for transcription initiation to proceed (Ghosh et al., 2010, Schumacher et al., 2006). Multiple intermediate states have been proposed to exist during transcription initiation (Davis et al., 2007). One such state has been captured between the AAA activator PspF (phage shock protein F) and RNAP-σ^54^-promoter DNA complex using an ATP hydrolysis transition state analog, ADP.AlFx (Bose et al., 2008, Chaney et al., 2001). These components provide a model system to capture the structural transitions for forming RPo.

In order to understand the transcription initiation process, which is highly dynamic and difficult to temporally resolve for structural studies, we took advantage of the bacterial σ^54^ system that forms stable RPcs. In this present work, using cryoelectron microscopy (cryo-EM) and single-particle analysis, we determined the structures of the RPc containing the promoter DNA from −35 to +28 with the characteristic fork junction structure of the RPc at −12/−11 (Guo and Gralla, 1998, Morris et al., 1994) and an intermediate complex (RPi) consisting of RNAP- σ^54^, the AAA domain of PspF (PspF_1-275_) in the presence of ADP.AlFx, and the promoter DNA. These structures represent snapshots of a complete bacterial RPc and its subsequent transcription intermediate complex, providing mechanistic insights into a number of key questions, including how (1) RNAP and σ factor engage with its promoter DNA in the RPc, (2) the DNA opening and transcription bubble formation are initiated, (3) the AAA activator interacts with RPc, and (4) conformational changes in σ^54^, DNA, and RNAP lead to DNA melting for transcription activation. Furthermore, we find unexpected and important roles for the AAA domains of the activators in the promoter DNA interactions and the associated transcription bubble formation.

## Results

### Structure of the RPc

**Figure 1:**
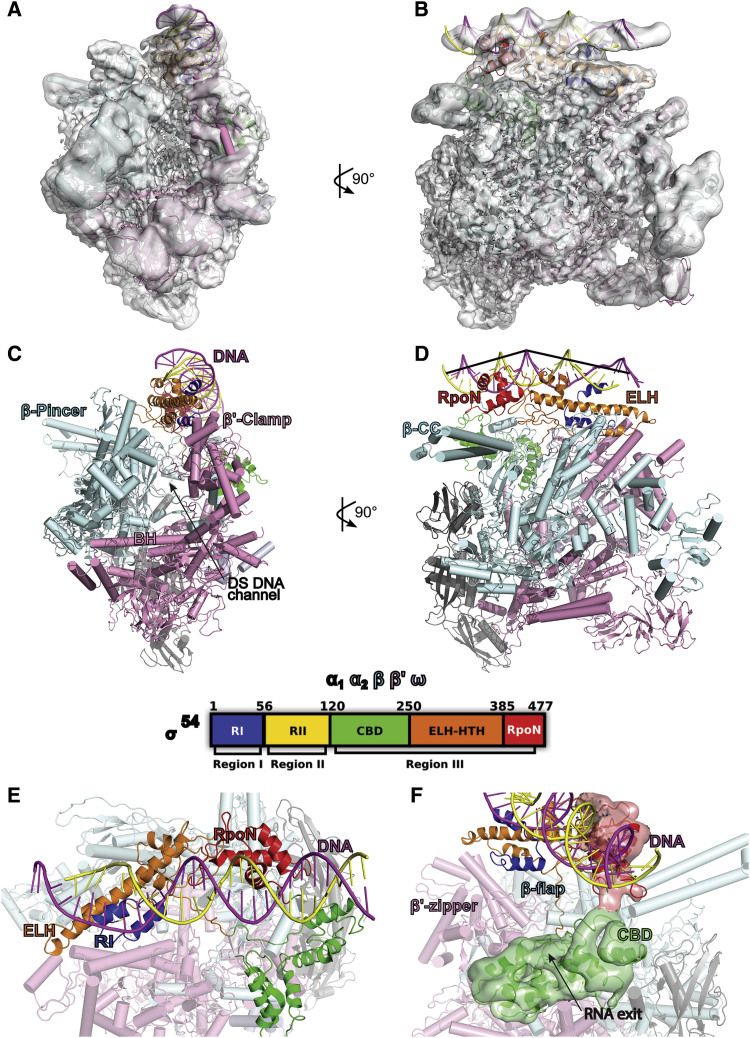
Cryo-EM Structural Model of RPc Consisting of RNAP-σ^54^-DNA (A and B) Cryo-EM map of RPc filtered to local resolution, with RNAP, σ^54^, and DNA structural models fitted in, viewed (A) into downstream DNA channel and (B) from β side. (C and D) RPc structural model in cartoon representations, viewed (C) into downstream DNA channel and (D) from β side. β pincer, β′ clamp, the bridge helix (BH), and downstream (DS) DNA channel labeled. (E) σ^54^ RI, ELH, and DNA (shown as cartoons) in RPc. σ^54^ domain organizations are shown. (F) Interactions between CBD and RpoN relative to the RNA exit channel. RNAP subunits are shown as cylinders. See also Figures S1 and S2and Table 1.

In order to understand how RNAP-σ^54^, and in general RNAP, is brought to the promoter DNA and how RPc assembles in a σ-dependent manner, we took advantage of the stable RPc formed by RNAP-σ^54^ and obtained the structure of the RPc consisting of RNAP, σ^54^, and promoter DNA using single-particle cryo-EM (Figures 1A, 1B, and S1). Previously, conformationally sensitive DNA footprinting results showed that DNA in RPc is distorted and displays base unstacking at the base pair downstream of the −12GC, and this distortion can be mimicked by a mismatch at −12/−11, to which RNAP-σ^54^ binds tightly (Morris et al., 1994). Our work presented here uses such a DNA containing a mismatch at −12/−11. Using the crystal structure of RNAP-σ^54^ (PDB: 5BYH) (Yang et al., 2015) filtered to 60 Å as a starting model and image processing in Relion (Scheres, 2012), the structure was refined to an overall resolution of 3.8 Å (Figures 1 and S1; Table 1) and has clear density for β, β′, two N-terminal domains of α subunit, ω, σ^54^, and DNA. The promoter DNA, although at lower resolution, is clearly visible and sits above the cleft formed between the large β and β′ subunits (also known as pincers) of RNAP (Figures 1A–1D and S1). The cleft contains the active center adjacent to the bridge helix that connects the two pincers and accommodates downstream DNA during transcription (Figures 1C and 1D) (Bae et al., 2015, Zhang et al., 2012b, Zuo and Steitz, 2015).

**Table 1:** Statistics of Cryo-EM Data Collection, Reconstructions, and Structure Refinements

	RPc^a^	RPi	RPi-RNAP^b^ Focus
**Data Collection**			

Total particles	312,669	480,143	480,143
Pixel size (Å)	1.06	1.06	1.06
Defocus range (μm)	−1.2 to 2.8	−1 to −3	−1 to −3
Voltage (kV)	300	300	300
Electron dose (e^−^ Å^−2^)	44	50	50

**Reconstruction Using RELION**			

Particles	80,810	79,355	89,387
Resolution (Å)	3.8	5.8	4.9

**Refinement**			

Resolution (Å)	3.8	5.8	–
MAP CC (whole map)	82.6	83.9	–
Map CC (around atoms)	72.5	63.4	–

**RMSD**			

Bond length (Å)	0.002	0.002	–
Bond angle (°)	0.608	0.564	–

**Ramachandran Plot**			

Preferred regions (%)	90.28	90.71	–
Allowed regions (%)	9.13	8.58	–
Outliers (%)	0.59	0.71	–

**Validation**			

All-atom clashscore	11.80	12.48	–
Rotamer outliers (%)	0.05	0.06	–
C-beta deviations	0	0	–

Map CC was calculated during Phenix real space refinement. RMSD, root-mean-square deviation.

a Note: disordered region in RPc: α-subunit (chain A), residues 1–4 and 238–329; α-subunit (chain B), residues 1–3, 160–171, and 239–329; β-subunit (chain C), residue 1342; β′-subunit (chain D), residues 1–14, 937–946, 1050–1056, 1068–1074, 1089–1096, 1127–1132, and 1377–1407; ω-subunit (chain E), residues 1 and 76–91; σ-subunit (chain M), residues 1–15, 50–117, and 474–477.

b Focus refinement of RNAP from RPi.

**Figure 2:**
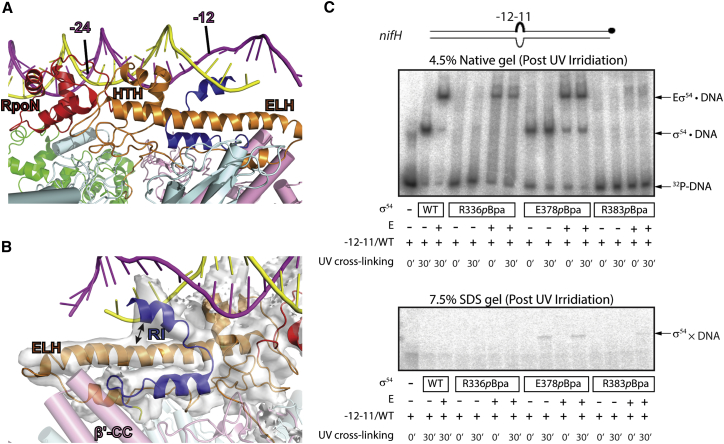
σ^54^ HTH Acts as a Major Anchor Point for Promoter DNA in RPc (A) Promoter DNA and its interactions with σ^54^. (B) Clear electron density is observed connecting RI and ELH (black arrow), suggesting a direct interaction between RI and ELH. (C) *p*Bpa mutants show that residues in HTH are crosslinked to promoter DNA. Top panel: native gel showing the ability of *p*Bpa mutants in forming RPc. Bottom panel: *p*BpaE378 and R383 within HTH are crosslinked to the early-melted *nifH* promoter DNA (with radiolabeling on the non-template strand; black circle) in the RNAP-σ^54^-DNA complex. E, RNAP enzyme. See also Figure S4.

The reconstruction reveals a dynamic complex with a well-defined RNAP core and less well-defined mobile elements of RNAP, the σ^54^, and the DNA (Figures 1A, 1B, and S1; resolution ranging from 3.0 to 7.5 Å). We obtained a structural model of RPc by first fitting the RNAP core of our previously determined crystal structure of RNAP-σ^54^ into the density, and subsequently moved domains of σ^54^ and built the DNA model (Figures 1A–1D). There is an alternative structural model of RNAP-σ^54^ (PDB: 5UI8) that was built and refined against our 3.8 Å crystallographic data of RNAP-σ^54^ (PDB: 5BYH; Yang et al., 2015) using a σ^54^ homology model derived from the recently determined crystal structure of *Aquifex aeolicus* (Aae) σ^54^ with the N-terminal domain deleted in complex with a promoter DNA (PDB: 5UI5) (Campbell et al., 2017). Although the overall domain structures remain unaltered and the alternative model does not contradict previous conclusions based on our crystal structure, there are differences between the two models in parts of σ^54^ (Campbell et al., 2017) where electron density was poor (Yang et al., 2015). We thus carefully reviewed both models and built and refined the crystal structural model (PDB: 5BYH) in combination with 5UI5 into the current density (see STAR Methods for details). In parts of RNAP, clear density is visible for side chains (Figure S2A); we built additional side chains into the structural model. Due to the lower resolution of the reconstruction and the ambiguity in the precise amino acid assignments in parts of σ^54^, we used the polyalanine trace of σ^54^ structural model from the 3.8 Å crystal structure. The density quality corresponding to the major RNAP-interacting domain, the core-binding domain (CBD), was sufficient to allow us to rebuild part of CBD (Figure S2B). The region between the region (R)III helix-turn-helix (HTH) domain and the C-terminal RpoN domain, which was missing in our previous crystal structure, was built using structural model of 5UI5 as a template. The region between CBD and extra-long helix (ELH)-HTH was of poor quality in both the crystal structure and the RPc density. Thus, we modeled the Cα trace using the model from 5UI5, which has well-defined structure for this region (Figures 1 and 2A) (Campbell et al., 2017). For DNA, a standard B-DNA was first fitted into the density corresponding to the upstream region (Figure 1B). Significant deformations from a B-form DNA occur downstream of −20 in the form of bending and stretching (Figures 2A). Individual DNA strands were subsequently adjusted to fit into the density. The DNA position and the RpoN domain orientation are aided by the NMR solution structure of *Aquifex aeolicus* (*Aae*)RpoN in complex with −24 promoter DNA (PDB: 2O8K) (Doucleff et al., 2007) and the *Aae*σ^54^ΔRI-DNA structure (PDB: 5UI5) (Campbell et al., 2017). Although the reconstruction precludes precise amino acid assignment, overall the σ^54^ model contains main chains of RI (∼16–50), CBD (120–260), ELH-HTH (∼320–386), and RpoN (∼410–473), and the DNA model includes DNA from ∼−33 to –5/−9. Specifying any detailed interactions involving specific side chains will require higher resolution structural data.

σ^54^ was proposed to impose a tight inhibition on RPc isomerization to RPo through blocking the DNA-RNA pathway used during transcription initiation and elongation, including occupying the downstream DNA channel and blocking the RNA exit (Yang et al., 2015). In the RPc structure, the N-terminal RI of σ^54^, which is the main determinant in σ^54^ binding to the activator protein, interacts with the ELH of σ^54^ RIII (Figure 1E), and together they form a barrier blocking DNA from entering the RNAP cleft for the RNA synthesis, in agreement with the previous crystal structure of RNAP-σ^54^ (Yang et al., 2015). Indeed, certain mutations in σ^54^, such as RI deletion or R336A (R336 resides within ELH), can bypass the requirement of activator proteins for transcription, provided the transcriptional bubble is pre-formed (Wang et al., 1997). There are direct interactions between ELH and RI (Figure 2B). Presumably, RI deletion or R336A mutation could disrupt these interactions, and thus relocate RI and ELH, releasing the inhibition imposed. The CBD interacts with β and β′ subunits as well as the very C-terminal tail of the σ^54^ RpoN domain in RPc (Figure 1F), and importantly, the CBD would block the exit for newly synthesized RNA (Figure 1F) (Yang et al., 2015). Furthermore, the cleft formed by the β and β′ pincers is too narrow toward the top (the narrowest point is <15 Å wide) for a double-stranded DNA to enter (Yang et al., 2015) (Figures 1A and 1C). RII was shown to occupy the downstream DNA channel and DNA/RNA channel (Yang et al., 2015). In RPc, we do not observe clear density in the downstream DNA channel, probably reflecting the flexible nature of RII. Together, these structural features support and extend the structural basis of σ^54^ inhibition on transcription (Yang et al., 2015).

σ^54^ recruits RNAP to the promoter DNA through binding to consensus promoter sequences at −24 and −12 (Buck and Cannon, 1992, Feklístov et al., 2014, Merrick, 1993). The RpoN domain directly interacts with the −24 promoter DNA (Figure 2A). In RPc, the σ^54^ RIII HTH interacts with DNA around −14 (Figure 2A). This arrangement is in agreement with chemical crosslinking and genetic data (Guo et al., 2000). The HTH domain forms an anchoring point for the promoter DNA onto RNAP, presumably through the interactions of σ^54^ ELH-HTH with both RNAP and the DNA (Figures 1D and 2A). Indeed, using UV-crosslinkable non-natural amino acids (Bpa), we found that Bpa substitutions of the highly conserved residues E378 and R383 within the HTH can be crosslinked to DNA, although R383pBpa variant prevented a stable RPc formation and only crosslinked to DNA in the presence of RNAP (Figures 2C) (Winkelman et al., 2015). Together, these observations support the idea that in RPc, ELH-HTH, which interacts with RNAP β and β′ subunits, acts as one of the major anchoring points for DNA interactions. Furthermore, the −24 interacting RpoN domain is anchored to RNAP through its interactions with σ^54^ CBD (Figure 1F). Together, the network of interactions between σ^54^ and RNAP as well as σ^54^ and DNA might explain the strict spacing requirement between −24 and −12 elements in all σ^54^ promoters (Buck, 1986, Merrick and Gibbins, 1985).

**Figure 3:**
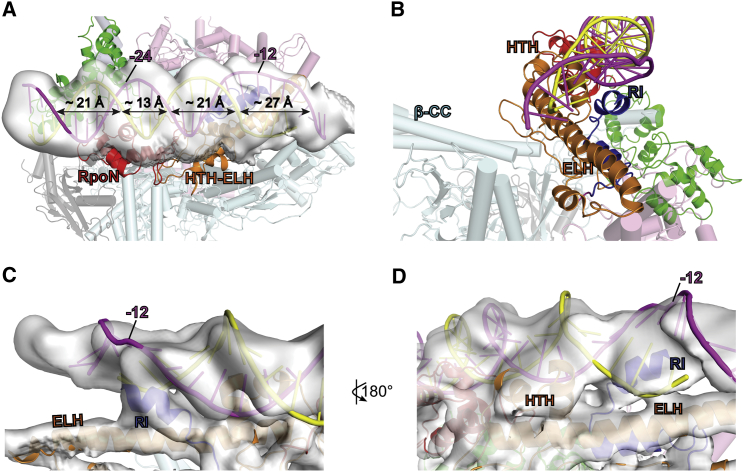
DNA Distortions and Interactions with σ^54^ RI and HTH in RPc (A) Distortion of DNA, especially downstream of −12, is evident. (B) σ^54^ RI (blue) in relationship to σ^54^ ELH (orange) and DNA (magenta and yellow). (C) Electron density map (surface) showing RI (blue) interacts with DNA. (D) Electron density map showing HTH (orange) interacts with one DNA strand (yellow). See also Figure S4.

In RPc, the promoter DNA is bent between −24 and −12 and there are significant distortions downstream of −12 compared to standard B-DNA (Figures 1 and 2A). Indeed, DNA is stretched with the minor groove widened to more than 25 Å and there is no clear density for a double-stranded DNA downstream of −9 (Figure 3A), suggesting a flexible nature of downstream DNA. The RI helix sits just underneath the DNA and above the ELH, coinciding with where significant DNA distortion starts (Figures 3A and 3B). There are direct interactions between the RI helix and both DNA strands downstream of −12 (Figure 3C), whereas HTH interacts with one of the strands in this region (Figure 3D). Thus, σ^54^ RI and HTH are positioned to stretch the DNA, thus helping to open up the transcription bubble.

### Structure of an RPi

**Figure 4:**
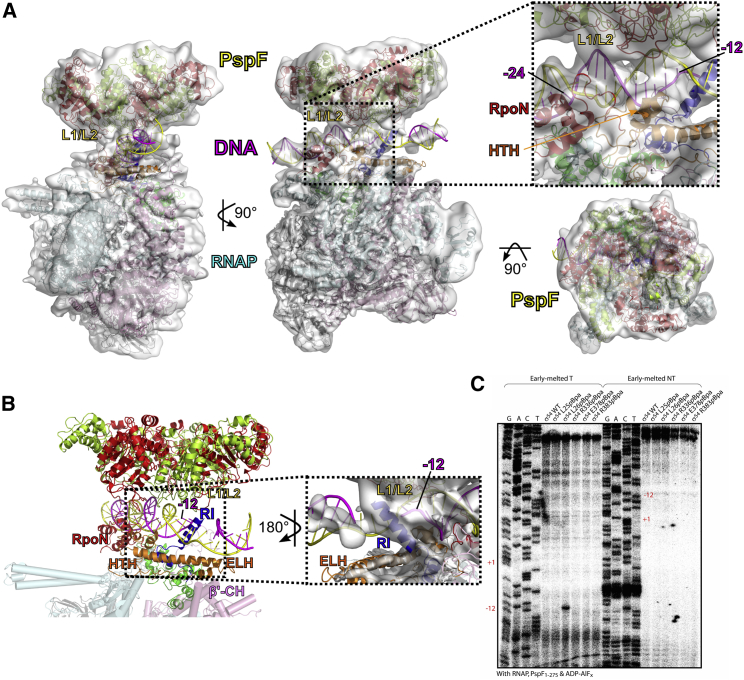
Three-Dimensional Reconstructions of RPi Consisting of RNAP-σ^54^-DNA-PspF (A) Cryo-EM map filtered to local resolution in three orthogonal views with coordinates fitted in. Inset shows the interactions between DNA and PspF. (B) PspF, σ^54^, and DNA in RPi. Inset shows the interactions observed in the electron density (shown as surface) between RI (blue), DNA (magenta and yellow), and PspF L1/L2 loops (red and green). (C) Crosslinking and primer extension assay in RPi show that L26 could be mapped to approximately −12 region of the template strand DNA in RPi. See also Figures S2 and S3and Table 1.

To investigate how transcription is activated and how the AAA activator interacts with RPc, we obtained a 3D reconstruction of an RPi consisting of RNAP, σ^54^, the same promoter DNA as in RPc, and the PspF AAA domain in complex with ADP.AlF_x_ (Figures 4 and S3). One of the most intriguing features that also posed significant challenges in the data analysis is the flexibility of the PspF relative to the RNAP (Figure S3). At least two distinct conformational classes were identified and the best class refined to 5.8 Å resolution (Figures 4A, S2D–S2F, and S3; Table 1), while RNAP was separately refined to 4.9 Å (Figure S3). The structural models of β, β′, two α N-terminal domains, and ω of RNAP were fitted into the 4.9 Å RNAP density (Figures 4A and S2D–S2F). The RNAP bridge helix is in a straight conformation and the front edge of the trigger loop/helix, which was shown to undergo structural transitions during nucleotide addition, remains as a flexible loop (Figure S2D). Additional density for σ^54^ is also clear. There is density above RNAP-σ^54^ and it can accommodate a hexameric PspF_1-275_ (Figure 4A). The density between the PspF and RNAP-σ^54^, despite the lower resolution, is clearly DNA (Figures 4A and S2F).

The limited resolution for the PspF hexameric ring prevented a precise modeling of individual PspF monomers into the electron density. We used the hexameric structure of NtrC1, another bEBP (PDB: 4LZZ; Sysoeva et al., 2013), as a starting model and manually adjusted the protomers. Each PspF protomer (Rappas et al., 2005) was then superimposed onto the NtrC1 protomer and AAA subdomains manually fitted into the density followed by rigid body refinements. The L1 loops, which are highly conserved signature loops in bEBPs and were disordered in the PspF crystal structure, were built using the L1 loops in NtrC1 as templates and manually positioned within the density. The σ^54^ model from RPc was positioned into the density and domains were then adjusted to best fit into the density. The density for ELH is clear, so ELH-HTH was fitted as a rigid body into the density (Figure S2F). There is also clear density for the RI helix (Figure S2F). The DNA model from RPc was first fitted in and adjusted manually followed by refinements (Figures S2D–S2F).

Despite the lower resolution of the PspF ring, it is clear that the PspF hexamer is highly asymmetric and resides above the DNA with L1/L2 loops facing DNA and RNAP-σ^54^ (Figures 4B). Importantly, there are significant interactions between PspF AAA domain and DNA, both with DNA backbones between −24 and −12 and directly in the groove downstream of −12 (Figure 4A and inset), where the transcription bubble starts. Despite the uncertainties in the exact position of the L1/L2 loops, it is clear that the interactions are mediated through L1/L2 loops that are main determinants for σ^54^ interactions, confirming previous data showing that the L1 loop is also closely associated with −12 DNA (Zhang et al., 2009, Zhang et al., 2012a). Similar to RPc, σ^54^ ELH-HTH and RI are also positioned proximal to the promoter DNA position around −12/−11 (Figures 2B and 4B, inset). Indeed when RPi formed with the *nifH* promoter DNA was UV irradiated to promoter crosslinking, and the approximate DNA position of the crosslink was mapped by subsequent separate primer extension reactions on each DNA strand, we found that L26 of RI helix could be mapped to approximately −12 template DNA strand in RPi (Figures 4C), in agreement with our structural model that RI helix is close to −12 DNA region. Significantly, there is density connecting RI helix to the PspF ring, in accordance with the established role of RI as the major interaction site for bEBP (Figure 4B, inset) (Bose et al., 2008). Immediately downstream, there is density for a single DNA strand followed by a double-stranded DNA (Figure 4B, inset), suggesting that a partial transcription bubble might have formed in RPi, in agreement with the observation in the RPc structure that the DNA strands are becoming distorted.

### Conformational Changes between RPc and RPi

**Figure 5:**
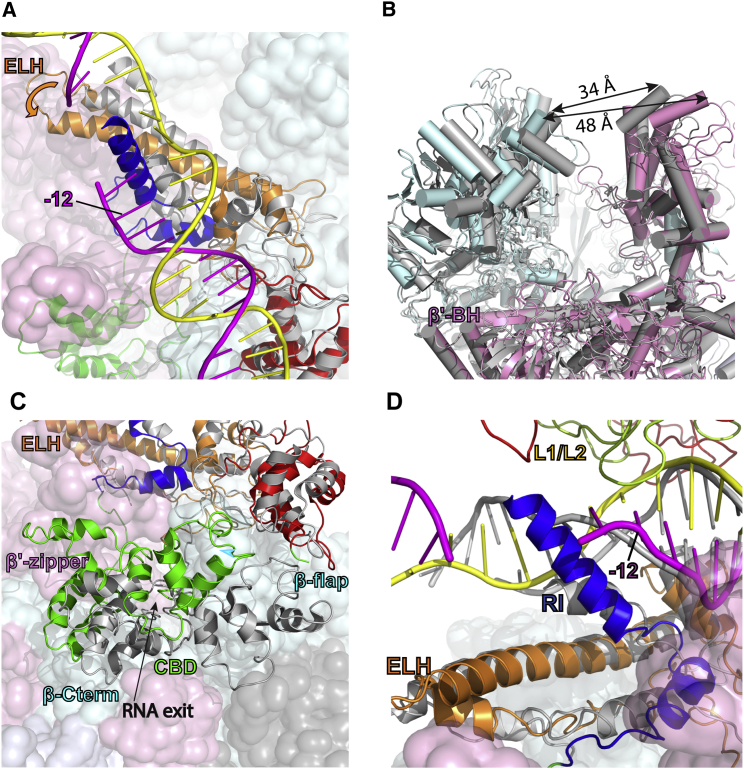
Comparisons between RPc, Displayed in Gray, and RPi, Displayed in Color (A) Comparisons of σ^54^ ELH in RPi (orange) and RPc (gray). ELH in RPi relocates away from the DNA entrance into the RNAP main channel. The DNA in RPi is shown. (B) Changes in β and β′ pincers and the RNAP cleft. (C) Changes in CBD relative to the RNA exit channel. (D) RI and DNA in RPc and RPi. In RPi, PspF L1/L2 (red and green) interact with DNA (magenta and yellow). RI (blue in RPi and gray in RPc) reaches up to interact with L1/L2, forming a wedge between DNA strands (magenta and yellow in RPi; gray in RPc). RNAP is shown as spheres, except in (B), while σ^54^ is shown as a cartoon. See also Figure S5.

In order to understand the transcription initiation process, we compared the RNAP-σ^54^ conformations between RPc and RPi by aligning their bridge helices, a key and structurally conserved global feature close to the RNAP active site (Figure 5). Significant conformational changes occur in σ^54^, β, and β′ upon activator interactions (Figure 5; Movies S1, S2, and S3), which explains how activator interactions induce changes that lead to RPo formation. First of all, the σ^54^ ELH and RI, which form an obstacle for the template DNA strand to enter the RNAP cleft in RPc, now move upstream in RPi, thus removing the blockage for DNA entry into the active cleft (Figure 5A; Movie S1). ELH and RI thus form a retractable gate for permitting promoter DNA entry. Second, the relocation of ELH also accompanies the changes in β and β′ pincer positions (Movie S1). The concerted movements of β and β′ pincers result in the widening of the cleft by ∼15 Å at the top in RPi compared to RPc (Figure 5B; Movie S2), in preparation for the loading of transcription bubble and downstream DNA into the RNAP cleft. This is in agreement with single-molecule FRET data, showing that although the clamp is dynamic and can adapt multiple conformations, there are more RNAPs with open clamp conformations in RPi compared to RPc (Chakraborty et al., 2012). Third, the σ^54^ CBD, which blocks the exit route for synthesized RNA in RPc, now starts to move away from the exit in RPi (Figure 5C; Movie S3). Finally, the interactions between PspF L1/L2 loops and σ^54^ RI form a wedge that could stabilize and propagate the strand separation initiated at −11/−10 by σ^54^ RI and HTH, helping with transcription bubble formation (Figure 5D). Interestingly, previous biochemical data show that RPi was competent in RNA synthesis provided the transcription bubble is pre-formed in the promoter DNA, in agreement with the observation that major obstacles in RNAP-σ^54^ for transcription have been removed in RPi, and that the transcription bubble formation/stabilization requires further actions of the activator protein (Burrows et al., 2010).

## Discussion

### DNA Distortions and Transcription Bubble Formation Are Initiated in RPc

The structures presented here reveal how the RNAP-σ^54^ engages with DNA, and subsequently with the activator, and how the interactions could proceed to cause further conformational changes that help the dissipation of the initially inhibited state. We show that upon interacting with RNAP-σ^54^, the promoter DNA, although remaining outside the RNAP cleft, is significantly distorted from a B-DNA (Figures 1, 2, and 3). There is no clear density downstream of −5/−9, suggesting a flexible nature, which could explain some of the deviations from B-DNA observed here. However, the stretching of the minor groove cannot be attributed to the flexible nature of a double-stranded DNA. The mismatch at −12/−11 is shown to mimic the distortion of DNA in RPc, and this distortion nucleates the melting (Morris et al., 1994); thus, this DNA represents the early melted DNA in RPc. The extensive interactions between σ^54^ and DNA support the idea that the interactions with RNAP-σ^54^ initiate and stabilize the distortions and nucleate the early melting. Interestingly, in the recently reported crystal structure of *Aae* σ^54^ with RI deleted (*Aae*σ^54^ΔRI) in complex with a promoter DNA, the DNA is shown to remain largely in B-form (Campbell et al., 2017) (Figure S4A). A number of factors could contribute to the differences: (1) In the RPc structure, the DNA contains a mismatch at −12/−11, which would cause local distortions in DNA. (2) The RPc and RNAP-σ^54^ structures show that RI plays important roles in interacting with ELH and DNA. The absence of RI and core RNAP in the *Aae*σ^54^ΔRI-DNA structure could thus influence the conformation of the DNA. Indeed, the distortion at −12 is only seen with the holoenzyme, not with the σ^54^ alone (Morris et al., 1994). (3) The RPc structure shows that σ^54^ RpoN, which interacts with the −24 region of the promoter DNA, also interacts with σ^54^ CBD, which in turn interacts with RNAP. ELH-HTH and RI, which interact with −12 and downstream DNA, directly interact with RNAP. This network of interactions among RI, ELH-HTH, RpoN of σ^54^, and RNAP would in turn impose constraints on the promoter DNA, inducing the deformations, including bending and stretching (Figure 2A). In the absence of RNAP and RI, as in *Aae*σ^54^ΔRI-DNA structure, these constraints are absent. The conformation of the protein could thus be determined by the B-form of the promoter DNA. By aligning the RpoN domains, the differences in conformation of ELH-HTH domain are obvious between the RPc structure and that of *Aae*σ^54^ΔRI-DNA, which coincide with the differences in DNA (Figure S4A). (4) In addition, the CBD, which was shown to interact with RNAP and nearly 50 Å away from ELH in the RPc structure, moved close to ELH-HTH in the *Aae*σ^54^ΔRI-DNA structure (Figure S4A), presumably due to the lack of both RNAP and σ^54^ RI in their structure. Despite these differences, we do not rule out that the conformation described in the *Aae*σ^54^ΔRI-DNA structure might partially represent the very initial stage of recruitment of the holoenzyme to promoter DNA.

Interestingly, RI and the HTH of σ^54^, which play key roles in DNA interactions that contribute to the DNA distortions, occupy positions relative to the promoter DNA similar to those occupied by region 2 and region 3 of σ^70^ in RPo (Figures 3 and S4C) (Bae et al., 2015, Zuo and Steitz, 2015). However, there are no structural or strict functional similarities between these σ^54^ and σ^70^ domains (Figure S4B). The latter interact with −10 elements and help with stabilizing the transcription bubble in RPo (Bae et al., 2015, Zhang et al., 2012b, Zuo and Steitz, 2015). In the *Thermus aquaticus* RNAP-σ^70^ (σ^A^) RPo structure, which contains a complete transcription bubble (Bae et al., 2015), E281 and R288 of σ^A^ region 3 are shown to recognize −14 GC bp through a polar interaction at C_−14_ template strand while R264 of σ^A^ region 2 is suggested to form H-bonds with the −14 bp of the non-template strand (Figure S4C). In σ^54^, there are highly conserved charged residues such as E378 and R383 in the HTH helix and there are several highly conserved Q and R in RI (Figures S4B and S4D). We speculate that some of these residues in σ^54^ could play similar roles in stabilizing the DNA strands once the transcription bubble is formed. We propose that through interactions with DNA −12 regions as observed in the RPc structure, σ^54^ RI and ELH-HTH help to initiate DNA melting. These regions could also stabilize the transcription bubble once formed, as observed in RPo of σ^70^ complex. It is possible that the residues in region 2 and region 3 of σ^70^ (σ^A^) could also be involved in initiating DNA distortions and early melting of the transcription bubble, which then spontaneously converts to an RPo, although the contacts have not been detected directly in an initiating RPi due to the transient nature of RPc in the σ^70^ system. Despite the lack of conservation in structures and sequences, it is possible that similar mechanisms in DNA melting operate through convergent evolution.

In the recently published yeast and human RNAPII RPc structures, the DNA bends so that the downstream DNA is just outside the RNAP main channel but DNA remains double stranded (Figure S5) (He et al., 2016, Plaschka et al., 2016). There are likely to be multiple conformational states between the initially formed closed complex and the stably formed RPc involving a distortion at −12/−11 (Friedman and Gelles, 2012). What we have captured is an early melted state in RPc while the RNAPII structures captured the initial closed complex. There are debates on whether the transcription bubble is formed outside the RNAP cleft, or whether double-stranded DNA is delivered first and the transcription bubble is then formed inside the cleft (Gries et al., 2010, Grünberg and Hahn, 2013). Our structure here suggests that transcription bubble formation can be at least initiated outside the RNAP cleft in the RNAP-σ^54^ system. Whether this is the case in other systems remains to be determined. It is possible that the eukaryotic system uses a different mechanism in DNA opening. Indeed, the upstream path of the DNA is different between RNAPII and bacterial RNAP-σ holoenzymes (Figure S5), reflecting the evolutionary divergence in promoter recognition and arrangement. Furthermore, although both RNAPII and RNAP-σ^54^ activations require an ATPase, TFIIH for RNAPII and bEBP for RNAP-σ^54^, their modes of action likely differ. bEBPs bind upstream of the TSS acting directly around −12 promoter region by removing the inhibition imposed by σ^54^ as well as helping with stabilizing the transcription bubble. The hexameric bEBP potentially allows multiple subunits to engage with RPc in a distinct set of actions. On the other hand, TFIIH binds downstream of the TSS and has been proposed to push DNA into the RNAP cleft through its helicase/translocase activities and the torsional strains in DNA promote DNA opening. However, a recent study shows that the ATPase activity of TFIIH subunit XPB is only required to relieve the auto-inhibition imposed by XPB, not DNA melting itself (Alekseev et al., 2017). It therefore remains to be seen how DNA melting and opening occur in RNAPII.

### The AAA Activator Plays an Active Role in DNA Melting as well as the Release of Inhibition Imposed by σ^54^

In RPi, the interactions of activator protein induce large conformational changes in σ^54^ and RNAP that contribute to the release of multiple inhibitions σ^54^ imposed on RNAP and enable DNA loading (Figure 5). Importantly, the AAA domain of activators directly interacts with the promoter DNA. Indeed, PspF L1/L2 loops interact with DNA downstream of −12 (Figure 4). Further, σ^54^ RI is positioned to insert itself between the two DNA strands to interact with PspF, and these interactions form a wedge between the DNA strands (Figures 4B, inset; Figure 5D), thus stabilizing the strand separation initiated at −11/−10 by σ^54^ RI and HTH, so helping with transcription bubble formation. Indeed, aromatic residues in region 2 of σ^70^ have been shown to play key roles in maintaining the double-strand/single-strand DNA junction of the transcription bubble (Fenton et al., 2000, Guo et al., 1999). Aromatic residues are absent in σ^54^ RI (Figure S4B), which could partially explain the requirement of activator proteins in DNA melting in σ^54^-dependent transcription. Interestingly, there is an invariant aromatic residue (F/Y) located at the tip of L1 loop (GAFTGA) of the activator protein (Lee et al., 2003, Rappas et al., 2005). Upon interacting with RNAP-σ^54^, the tip of the L1 loop could be positioned to interact with DNA (Figure 4B, inset; Figure 5D); the current resolution of the reconstruction prevents the loops from being accurately located. In promoter DNA binding assays using ADP.AlFx, the substitution of F by Y in PspF L1 loop resulted in the loss of stable binding to DNA that has a mismatch at −12/−11 (Zhang et al., 2009), further supporting the idea that F85 might be directly involved in DNA interactions. Chemical crosslinking data show that residues in L1 of PspF can be directly crosslinked to DNA (Zhang et al., 2012a). This lends support to the tentative idea that activator could thus contribute an aromatic residue in DNA interactions and transcription bubble formation/stabilization. The importance of the aromatic residue is shown by mutational studies; F-to-Y mutation supported partial transcription while F-to-A abolished its functionalities (Zhang et al., 2009). F-to-W mutation failed to interact with σ^54^, probably because this mutation affects the conformation of L1 that harbors the main determinants for σ^54^ interactions. The σ^54^ bypass mutants, such as RI deletion or R336A, can only support transcription in the absence of activator provided the transcriptional bubble is pre-formed (Wang et al., 1997), further supporting a role for the activator and RI in transcription bubble formation and/or stabilization.

### Unique Activator Positions and Mechanisms of Activators

**Figure 6:**
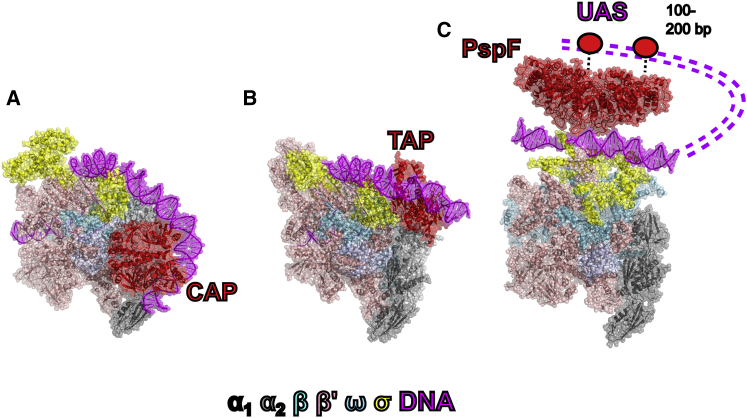
Comparison of the Bacterial RNAP Activator-Bound Complexes (A) σ^70^ class I activator CAP complex. (B) σ^70^ class II activator TAP complex. (C) bEBP activator complex as observed here. Shown for illustrative purposes are upstream activator sequences (dashed lines) and DNA binding domains of bEPB (ellipses). Activators are shown in red, σ factors in yellow, and DNA in magenta. See also Figure S6.

The intermediate complex structure presented here shows a unique position of bEBP compared to other activator-bound RNAP structures, which explains some of their functional differences. For the classic σ^70^ class I and class II activators such as CAP/CRP, the activator binds to upstream DNA close to the promoter region through interactions with α C-terminal domain, adjacent to the RNAP (Figure 6) (Feng et al., 2016, Hudson et al., 2009), to increase association of RNAP-σ^70^ with promoter DNA. On the other hand, bEBPs bind to DNA remotely from promoter DNA and function through actively remodeling both promoter DNA and RNAP-σ^54^, which can only be achieved through interactions made from being directly above the RNAP cleft where the promoter DNA is bound. The bEBPs thus interact with RNAP-σ^54^ via DNA looping to sandwich promoter DNA and do not utilize α C-terminal domain in its interactions (Figure 6).

Nucleotide binding and hydrolysis by the activator proteins are required for transcription activation. Previously, we have shown that nucleotide-bound states are directly correlated to the conformation of L1 and L2 loops, thus controlling the interactions between activator and RNAP-σ^54^ as well as remodeling the interactions (Rappas et al., 2005, Rappas et al., 2006). Here our structures show that the interactions also include contacts to promoter DNA. The activator belongs to the AAA ATPase family, which includes NtrC1, E1, and Rho helicases that showed asymmetric arrangement of protomers within the hexamer. A sequential mechanism has been proposed for E1 and Rho to processively remodel their substrates (Enemark et al., 2000, Thomsen and Berger, 2009). PspF displays a similar asymmetric arrangement to NtrC1, E1, and Rho (Figure S6). Although bEBPs might not require continuous hydrolysis, nucleotide binding and hydrolysis among some subunits within the hexamer may induce changes in these and other subunits required for the series of substrate remodeling events, including DNA distortions and transcription bubble formation as well as conformational changes in RNAP-σ^54^ (Joly et al., 2006).

### Mechanisms of Transcription Initiation

The RPc and RPi structures reported here allow us to speculate that in RNAP-σ^54^ system, the promoter DNA distortion and transcription bubble formation are initiated in RPc and that transcription bubble formation involves an active DNA melting step. Interestingly, we observe large conformational changes in RNAP—for example, the clamp opening—during RPc to RPi transition. While comparing RNAPs in the RPc structure with that of RNAP-σ^70^ RPo, the conformational changes are modest. Our studies thus suggest that during transcription initiation, large conformational changes in RNAP are required for DNA delivery and transcription bubble formation in the RNAP active center before the RNAP returns to a conformation more similar to RPc once the DNA is delivered and RPo is formed. These conformational changes can occur spontaneously in σ^70^-dependent transcription but require ATP-dependent activators in σ^54^-dependent transcription due to the structural inhibitions imposed by σ^54^. The structures presented here also suggest that the conformations for an intermediate state cannot be inferred from that of RPc or RPo alone. Some clear parallels also exist in eukaryotic transcriptional machineries. Although only modest conformational changes have been observed in RPo compared to RPc in RNAPII (Plaschka et al., 2016), the RNAP clamp opening is different in the yeast and human RNAPII RPc structures, suggesting that clamp opening could be a common theme in transcription initiation. The human and yeast RPc structures might represent slightly different functional states during transcription initiation, although no RPi structure has been captured for RNAPII (He et al., 2016, Plaschka et al., 2016). In yeast and human RNAPII RPo complex, TFIIB, TFIIE, and TFIIF play key roles in interacting with −10 DNA and maintaining the transcription bubble. In bacteria, region 2 and 3 of σ^70^ play similar roles (Figures S4C, S4D, and S5), while work presented here suggests that RI and ELH-HTH of σ^54^ could play an active role in initiating DNA melting. It will be interesting to see if these and other eukaryotic factors also play an important role in DNA distortion and helping with initiating transcription bubble formation. It is plausible that bacterial and eukaryotic systems share a common core mechanism in DNA melting and transcription stabilization during transcription initiation, although systems have diverged to impose different levels of regulation.

## STAR★Methods

### Key Resources Table

**Table:** 

REAGENT or RESOURCE	SOURCE	IDENTIFIER
**Bacterial and Virus Strains**		

*E. coli* 10-beta	NEB	#C3019H
*E. coli* BL21(DE3)	NEB	#C2527
*E. coli* BL21-GOLD (DE3)	Agilent Technologies	# 230132

**Chemicals, Peptides, and Recombinant Proteins**		

Plasmid pGEMABC (N-terminal 6 × His tag, encoding full length of *E. coli* rpoA, rpoB and rpoC)	(Yang et al., 2015)	addgene #45398
Plasmid pACYCDuet-omega (without tag, encoding full length rpoZ of *E. coli*)	(Yang et al., 2015)	N/A
Plasmid pET28b-σ54 (N-terminal 6 × His tag, encoding full length of σ54 from K. pneumoniae M5A1	(Yang et al., 2015)	N/A
Plasmid pET28b-PspF1-275 (N-terminal 6 × His tag, encoding residues 1-275 of PspF from *E. coli*)	(Rappas et al., 2005)	N/A
Plasmid pMKC28-nifH	(Chaney and Buck, 1999)	N/A
pET28b-σ54 R336 pBpa	This work	N/A
pET28b-σ54 L25 pBpa	This work	N/A
pET28b-σ54 E378 pBpa	This work	N/A
pET28b-σ54 L26 pBpa	This work	N/A
pET28b-σ54 E383 pBpa	This work	N/A
H-p-Bz-Phe-OH (pBpa)	Bachem	F-2800.0005

**Deposited Data**		

Close complex RPc	This work	PDB: 5NSR, EMD: 3695
Intermediate complex RPi	This work	PDB: 5NSS, EMD: 3696
Local focused refined RNAP map from RPi	This work	EMD: 3697

**Oligonucleotides**		

nifH σ54 promoter DNA template strand ACATGAATGCGCAACAGCATGCGCGCCCAGGGCTGATCGTGCAAAAGTCGTGCCAGCCGTC	This work	N/A
nifH σ54 promoter DNA non-template strand GAGACGGCTGGCACGACTTTTGCCAGATCAGCCCTGGGCGCGCATGCTGTTGCGCATTCATGT	This work	N/A
nifH promoter DNA WT template −60 to +60 for primer extension GTTGTTTAAGCTATTTCGGTTGTTCGGACACATGAATGCGCAACAGCATGCGCGCCCAGGGCTGATCGTGCAAAAGTCGTGCCAGCCGTCTGAAATAAAACTACTCGGCTTTCTTTCAGA; nifH promoter DNA early-melted non-template −60 to +60 for primer extension TCTGAAAGAAAGCCGAGTAGTTTTATTTCAGACGGCTGGCACGACTTTTGCcaGATCAGCCCTGGGCGCGCATGCTGTTGCGCATTCATGTGTCCGAACAACCGAAATAGCTTAAACAAC	This work	N/A
32P-labeled forward primer for primer extension TCTGAAAGAAAGCCGAGTAGTTTTA	This work	N/A
32P-labeled reverse primer for primer extension GTTGTTTAAGCTATTTCGGTTGTTC	This work	N/A

**Software and Algorithms**		

COOT	(Emsley et al., 2010)	https://www2.mrc-lmb.cam.ac.uk/personal/pemsley/coot/
Relion1.4	(Scheres, 2012)	http://www2.mrc-lmb.cam.ac.uk/relion/index.php/Main_Page
Chimera	(Pettersen et al., 2004)	https://www.cgl.ucsf.edu/chimera/
Phenix real_space_refine	(Afonine et al., 2013)	https://www.phenix-online.org/documentation/reference/real_space_refine.html
Refmac	(Brown et al., 2015)	N/A
Motioncorr	(Li et al., 2013)	http://cryoem.ucsf.edu/software/driftcorr.html
Gautomach	N/A	http://www.mrc-lmb.cam.ac.uk/kzhang/
CTFFIND4	(Rohou and Grigorieff, 2015)	http://grigoriefflab.janelia.org/ctffind4

### Contact for Reagent and Resource Sharing

Further information and requests for resources and reagents should be directed to and will be fulfilled by the Lead Contact, Xiaodong Zhang (xiaodong.zhang@imperial.ac.uk).

### Method Details

#### Sample preparation

*E. coli* RNA polymerase and *K. pneumoniae* σ^54^ were expressed and purified as described previously (Yang et al., 2015). The σ^54^ holoenzyme was formed by incubating RNAP with a four-fold excess of σ^54^ before size exclusion chromatography using a Superose 6 10/300 column (GE Healthcare) equilibrated in buffer S6 (10 mM Tris-HCl pH 8.0, 150 mM NaCl, 10mM MgCl_2_ and 5% glycerol). The same 63 bp nucleic acid scaffold was used for both the closed (RPc) and intermediate (RPi) complexes. This consists of the sequence from a *nifH* σ^54^ promoter from positions −35 to +28 (relative to TSS at +1) with a mismatch at −11/-12 to mimic initial fork junction formation (template strand 5′-ACATGAATGCGCAACAGCATGCGCGCCCAGGGCTGATCGTGCAAAAGTCGTGCCAGCCGTCTC-3′, non-template strand 5′-GAGACGGCTGGCACGACTTTTGCCAGATCAGCCCTGGGCGCGCATGCTGTTGCGCATTCATGT-3′).

The RPc was formed by incubating purified holoenzyme with a 1.2 times molar excess of DNA before size exclusion chromatography using a Superose 6 10/300 column (GE Healthcare) equilibrated in buffer-EM (10 mM Tris-HCl pH 8.0, 150 mM NaCl, 10 mM MgCl_2_).

*E. coli* PspF residues 1-275 (PspF_1-275_) was expressed and purified as described previously (Rappas et al., 2005). The RPi was formed by first incubating purified holoenzyme with a 1.2 molar excess of DNA. The holoenzyme-DNA complex was then incubated with six-fold excess of PspF_1-275_, and large excesses of ADP and NaF at 37°C for 5 min in STA buffer (25 mM Tris-acetate pH 8.0, 8 mM Mg-acetate, 10 mM KCl and 1 mM DTT). AlCl_3_ was then added before a further 20 min incubation. The trapped intermediate complex was then purified by Superose 6 size exclusion chromatography using buffer-EM.

#### Electron microscopy

The RPc samples were applied at a concentration of 0.5 mg/ml to R1.2/1.3 holey carbon grids (Quantifoil) whereas the RPi samples were at a concentration of 0.3 mg/ml with R2/2 holey grids. 3 μL of samples were applied to each grid, which was blotted and vitrified using a Vitrobot Mark IV (FEI) at 4°C and 100% humidity. Data were collected at eBIC (Diamond Light Source, UK) on a Titan Krios operated at 300 keV using a K2 Summit direct electron detector (Gatan) and a pixel size of 1.06 Å/pixel. Data collection was carried out automatically using EPU software (FEI).

For the RPc, a total of 1415 movies were collected with a defocus range of −1.2 μm to −2.8 μm. Each movie was collected with an 8 s exposure with a total dose of 44 e^-^/Å^2^ fractioned into 32 frames. The total intermediate complex dataset was 3858 movies from a 10 s exposure fractioned into 40 frames with a total dose of 50 e^-^/Å^2^.

#### Image processing

Both datasets were processed using similar approaches initially. Individual frames were aligned using motioncorr (Li et al., 2013), CTF parameters were estimated using CTFFIND4 (Rohou and Grigorieff, 2015) and reference-based particle picking was performed using Gautomatch. All other processing steps were performed with RELION 1.4 (Scheres, 2012). Resolution estimates were carried out inside RELION using the gold-standard Fourier shell correlation (FSC = 0.143) criterion (Rosenthal and Henderson, 2003). Local resolution estimate was performed using RELION 2.0. Particles were extracted into boxes of either 256x256 pixels (RPc) or 272x272 pixels (RPi). Initial 2D classification was used to remove incorrect particles for downstream processing.

RPc dataset image processing procedure is summarized in Figure S1. The initial model was generated using the crystal structure of RNAP-σ^54^ low-pass filtered to 60 Å. The best particles were refined against this model using RELION’s 3D auto-refine procedure before performing 3D classification without alignment of the particles. The best class containing all domains for RNAP and σ^54^ as well as DNA was then refined further. RELION’s particle polishing procedure was used to correct for particle movement and to perform per-frame B-factor weighting. The output ‘shiny’ particles were re-refined resulting in the best map to an overall resolution of 3.8 Å.

Processing procedure of the RPi dataset is illustrated in Figure S3. Briefly, the best particles following initial 2D classifications were refined against the low resolution RPi model without DNA (EMD: 1566). Particles were then subject to 2D classification without alignment to further remove junk and badly aligned particles. Particle polishing was performed before separating out different conformations using 3D classification. The best class showing good density for RNAP, PspF_1-275_, σ^54^ and DNA was refined to an overall resolution of 5.8 Å (Figure S3). To improve the resolution of the core RNAP, focused refinements around RNAP alongside partial signal subtraction (Bai et al., 2015) were performed. Following the first round of 3D classification, those particles that correspond to complexes with the best RNAP features (but different PspF ring conformations) were subject to procedures that remove signals that correspond to those of PspF. The resulting particles (with PspF density subtracted) were combined and then re-classified into three 3D classes. The best class corresponding to 60% of the new particle set was then refined to an overall resolution of 4.9 Å.

#### Choice of Input Coordinates Sets, Model Building and refinement

Command scripts for map conversion and structure refinement in Refmac (Brown et al., 2015) were provided by Garib Murshudov (MRC-LMB). For RPc model building, the crystal structure of RNAP-σ^54^ (PDB: 5BYH; Yang et al., 2015) was used as a template for initial global docking in chimera (Pettersen et al., 2004). The crystal structure of *Aquifex aeolicus* (Aae) σ^54^ with promoter DNA (PDB: 5UI5; Campbell et al., 2017) were also used to guide the building of σ^54^. An alternative structural model for RNAP-σ^54^ (PDB: 5UI8) (Campbell et al., 2017) was built into the same 3.8 Å diffraction data (PDB: 5BYH; Yang et al., 2015). One of the main differences between these two structural models lies in Region III. In and around the ELH (residue ∼320-360), the amino acid register differs by up to 5. The difference largely arises from the loop regions surrounding the ELH due to the poor electron density in those regions. We were aware of the limitations of our crystal structure derived from 3.8 Å crystallography data and thus in our original publication (Yang et al., 2015), we almost exclusively referred to domains and regions instead of individual residues. There are merits in the alternative model as it was guided in its building by two structures, our original crystal structure (PDB: 5BYH) and the homology model derived from *Aae*σ^54^-DNA structure (PDB: 5UI5) (Campbell et al., 2017). We used Se-Met derived σ^54^ and used the Se-Met sites obtained from anomalous difference map to help with amino acid register (Yang et al., 2015). In the region preceding the ELH, in 5BYH, Met306 is close to a Se-Met site, while this is not the case in 5UI8 (Figure S2; Yang et al., 2015). We have thus used 5BYH as a template for RPc but with reference to the *Aae*σ^54^ model (PDB: 5UI5) for regions that were missing or poorly resolved in 5BYH but are present in 5UI5. However, for regions that remain poorly defined, we have either removed side chains or used UNK as amino acid identifiers to indicate that in these regions, the amino acid registers are ambiguous. The DNA structure was first generated as an ideal double-stranded B-DNA and then manually fitted into the density in Coot (Emsley et al., 2010).

For RPi model building, RNAP-σ^54^ and DNA were built using RPc as templates and manually adjusted to fit into density, while the hexameric ring of PspF was built using NtrC1 structure (PDB: 4LZZ; Sysoeva et al., 2013) as a starting model and manually adjusted to fit into the density. Each PspF protomer (PDB: 2BJV; Rappas et al., 2005) was then superimpose into the NtrC1 protomer individually and manually fitted into the density in Coot (Emsley et al., 2010). For RNAP, the 4.9 Å map from focused refinement was used in building.

For both RPc and RPi model refinements, jelly body and reciprocal-space refinement using Refmac were first performed to maintain geometric restraints (Brown et al., 2015). Afterward, real_space_refinement was carried out in Phenix (Afonine et al., 2013) to correct Ramachandran and rotamer outliers. Finally, Phenix was run in Atomic Displacement Parameters (ADP or B-factors) mode to assign atom B-factors and to generate the final model statistics (Table 1). Visualizations were carried out in PyMol or Chimera (Pettersen et al., 2004).

#### pBpa-based UV cross-linking assay

Reactions were performed in 10 μl volumes and supplemented with 1 μM σ^54^-*p*Bpa variant, ± 0.3 μM core RNAP, and 50 nM ^32^P-labeled *nifH* early-melted promoter DNA (−12-11/WT) in STA1 buffer (2.5 mM Tris-acetate pH 8.0, 10 mM KCl, 1 mM DTT, 3.5% (w/v) PEG 8000) at 37°C for 10 min. Reaction mixtures were UV-irradiated at 365 nm on ice for 0 or 30 min, analyzed on both native and SDS-PAGE gels, and detected by a Fuji PhosphoImager.

#### Primer extension assay

Reaction were performed in 20 μl volumes containing: 75 nM *nifH* early-melted promoter DNA (−12-11/WT), 400 nM His-tagged σ^54^-*p*Bpa variant, 200 nM core RNAP, 2.4 μM PspF_1-275_, 4 mM ADP, 5 mM NaF and 0.4 mM AlCl_3_ in STA buffer at 37°C for 10 min, and then UV-irradiated at 365 nm on ice for 40 min. After cross-linking, the reaction mixtures were added to 20 μl of Ni-NTA magnetic agarose slurry (QIAGEN) equilibrated in high salt buffer (40 mM Tris-HCl pH 8.0, 1 M NaCl) for enrichment. After incubation at room temperature for 10 min, the supernatant was removed and the beads were washed twice in high salt buffer before the final resuspension in 50 μl of 1x Taq Buffer (10 mM Tris-HCl pH 8.0, 50 mM KCl, 1.5 mM MgCl_2_). Four μl of the enriched beads were used as a template for primer extension in a 12.5 μl reaction volume containing: 1 unit of Phusion High-Fidelity DNA polymerase (New England BioLabs), 1 mM ^32^P-labeled forward or reverse primer, 1.25 mM of each dNTP, 5% DMSO in 1x HF buffer (New England BioLabs). Extension products were amplified by PCR (2 min at 95°C and 19 cycle of 30 s at 95°C, 30 s at 55°C and 45 s at 72°C). Four μl of each PCR product were mixed with an equal volume of footprinting dye (3 mg xylene cyanol, 3 mg bromophenol blue, 0.8 mL of 250 mM EDTA, 10 mL of deionized formamide) and resolved on a 10% Urea gel at 50 W for 2 hr 20 min. The DNA ladder was produced using Thermo Sequenase Cycle Sequencing Kit (Affymetrix) on a pMKC28-nifH plasmid according to the manufacturer’s protocol.

### Quantification and Statistical Analysis

Please see Table 1 for quality of 3D reconstructions and models.

### Data and Software Availability

The accession numbers for the cryo-EM reconstructions of the RPc, RPi, and RNAP focused map within RPi reported in this paper are EMD: 3695, 3696, and 3697. The structural models of RPc and RPi reported in this paper are deposited in the PDB under accession numbers PDB: 5NSR and 5NSS.

## Author Contributions

X.Z. and M.B. designed and supervised the study, R.G. and V.C.D. carried out the cryo-EM studies, and F.Y. built and refined the models, while N.Z. performed the biochemical experiments. X.Z. and M.B. prepared the manuscript with input from all authors.

## Supplemental Information

Document S1. Figures S1–S6

Movie S1. Morphing between RPc and RPi Structures, Related to Figure 5 For clarity, only the structures of RNAP-σ^54^ are shown. Viewed from the top showing the σ^54^ ELH and β’-clamp relocations. cyan- β, light pink – β’, blue-σ^54^ RI, orange – ELH-HTH, green – CBD, red – RpoN. For reference, a transcription bubble (magenta/yellow) from 4YLN, by aligning bridge helices, was also displayed.

Movie S2. Same as Movie S1, Related to Figure 5 Viewed into the downstream DNA channel showing the cleft opening.

Movie S3. Same as Movies S1 and S2, Related to Figure 5 Viewed from the β’ side showing the relocation of CBD.

Document S2. Article plus Supplemental Information
