# Supplementary material for: Structures of RNA Polymerase Closed and Intermediate Complexes Reveal Mechanisms of DNA Opening and Transcription Initiation
Source: Mol Cell. 2017 Jul 6;67(1):106–116.e4. doi: 10.1016/j.molcel.2017.05.010 (PMC5505868; doi:10.1016/j.molcel.2017.05.010)
Supplement: Document S1. Figures S1–S6 [file mmc1.pdf]

Molecular Cell, Volume *67*

Supplemental Information

Structures of RNA Polymerase Closed and Intermediate Complexes Reveal Mechanisms of DNA Opening and Transcription Initiation

Robert Glyde, Fuzhou Ye, Vidya Chandran Darbari, Nan Zhang, Martin Buck, and Xiaodong Zhang

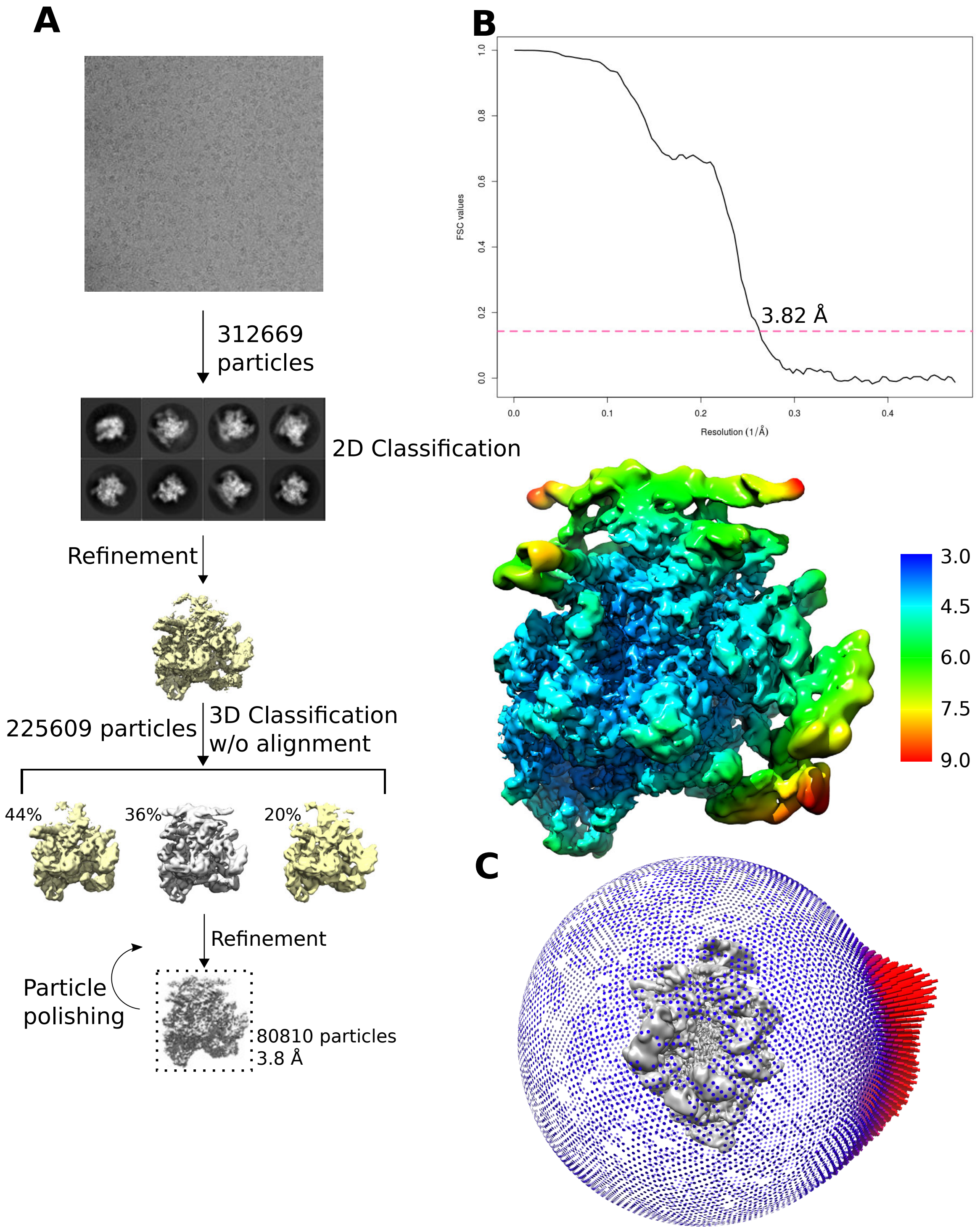

Figure S1, related to Figure 1. RPc EM data quality and image processing. (A). A representative micrograph, 2D classes and image processing flowchart, **(B).** FSC curve and local resolution map (in Å) calculated using RELION 2.0. **(C).** angular distribution of the particles used in the final reconstruction.

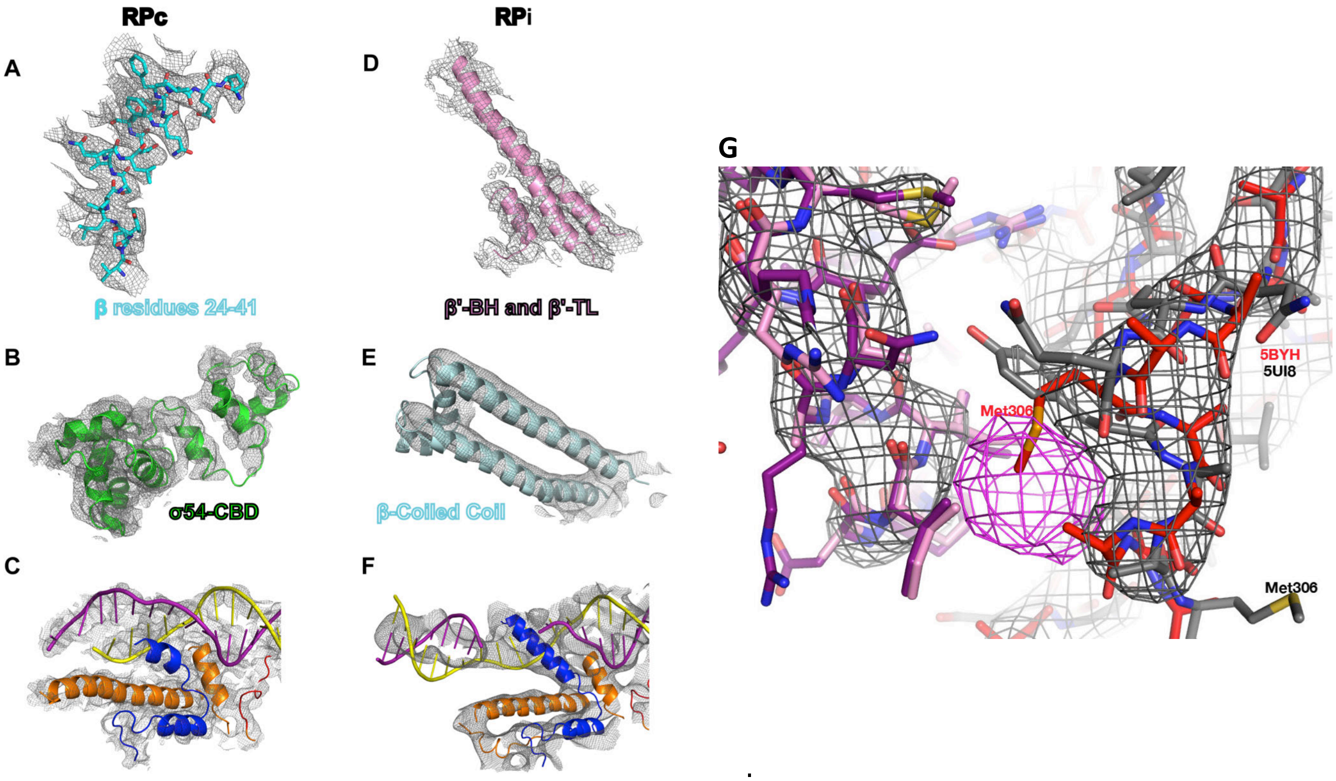

Figure S2, related to Figures 1-4 and STAR Methods. Examples of electron density and models that are fitted in. (A-C) RPc and **(D-F)** RPi showing the regions in RNAP where clear side chain density is visible (**A**), where a whole domain can be moved in as a rigid body (**B and E**), parts of σ^{54} and DNA where only main chain can be traced (**C-F**). **(D)** also shows the β'-bridge helix and trigger loop in RPi. **(G)** Differences in the structural models of 5BYH and 5UI8 around Met306. Anomalous Fourier difference map (DANO) in magenta calculated from Se-Methionine single anomalous diffraction dataset collected for the RNAP-σ^{54} holoenzyme containing Se-Met derivitized σ^{54} (Yang et al., 2015) using the original phases determined by molecular replacement using the RNAP model from 4YG2. The DANO map is contoured at 6.5σ clearly identifies the Se-Met site (the peak for this site is at 10σ), which is in close proximity to Met306 side chain in 5BYH (red) while far from that in 5UI8 (grey). Also shown is the weighted 2Fo-Fc map (in grey) calculated using phases from low resolution refinement of 5BYH in Refmac. β' subunits are in pink (5BYH) and purple (5UI8)

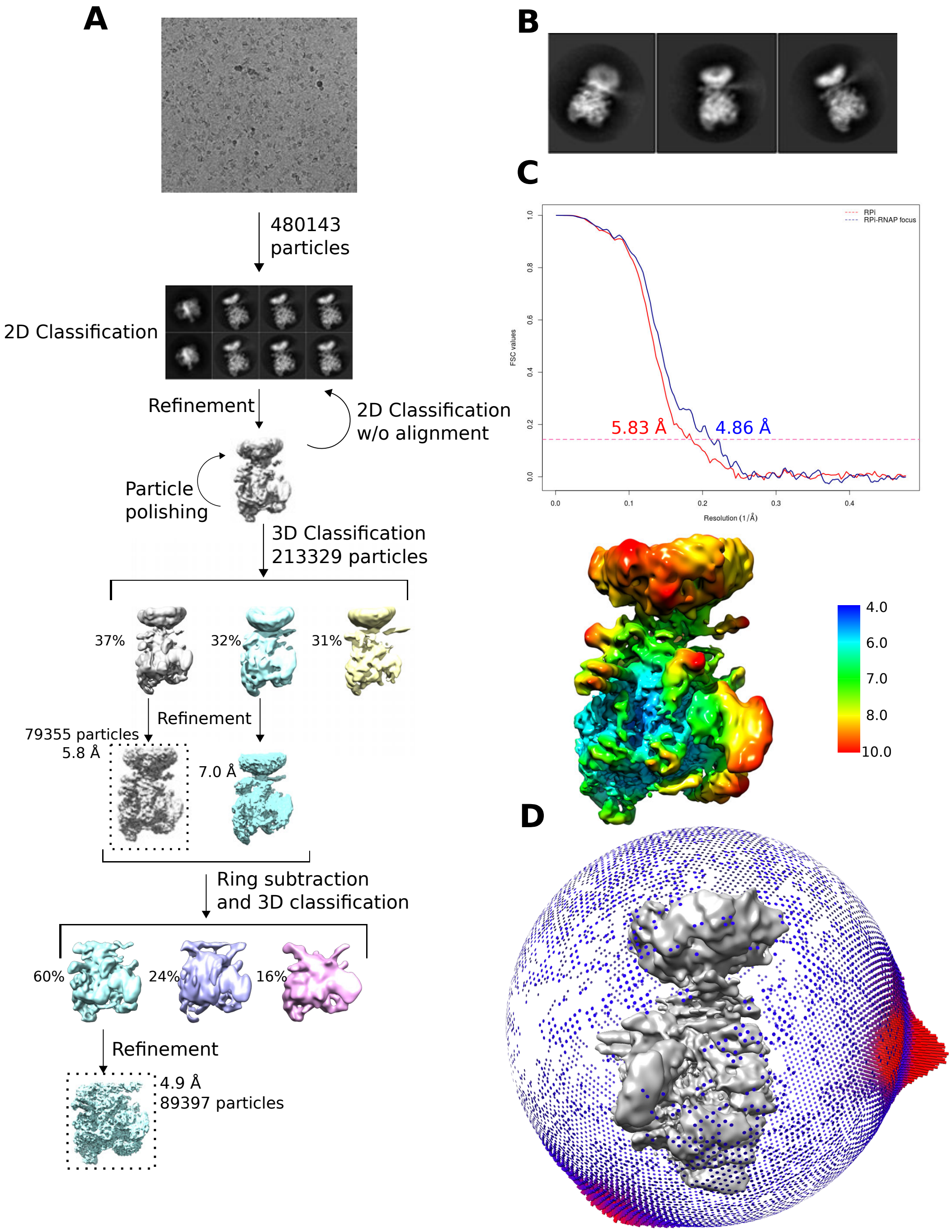

Figure S3, related to Figure 4. RPi cryo-EM data quality and image processing. (A). A representative micrograph, 2D classes and image processing flowchart, **(B).** 2D classes showing the flexibility of the hexameric ring (top) relative to the RNAP (bottom). (**C**) FSC curves for RPi (red) and the focused refinement on RNAP (blue) and local resolution map for RPi (in Å). **(D).** augular distribution of particles used in the final RPi reconstruction.

A

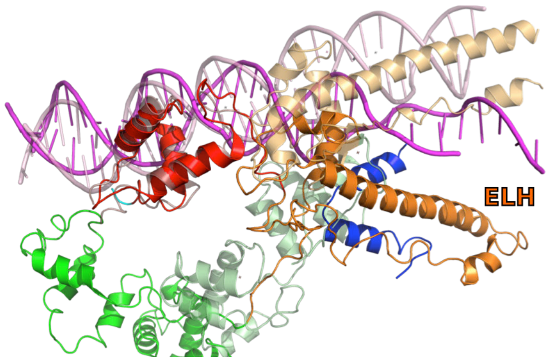

B

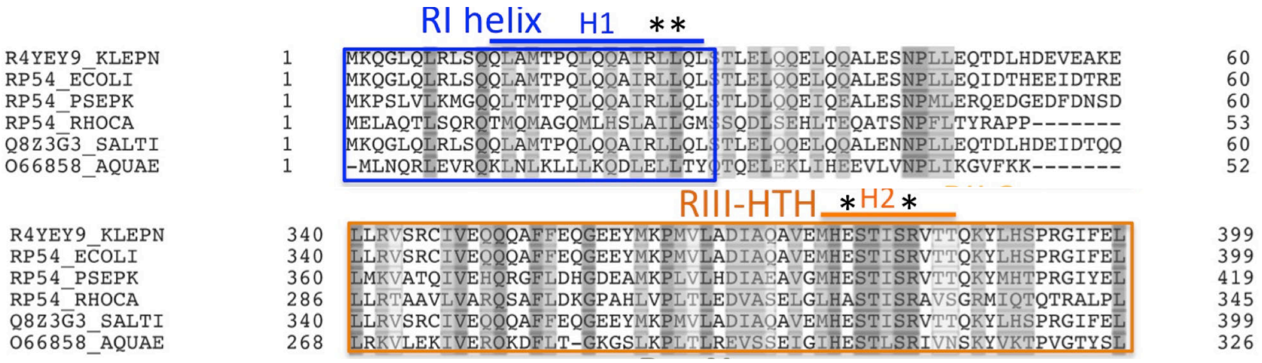

C **D**

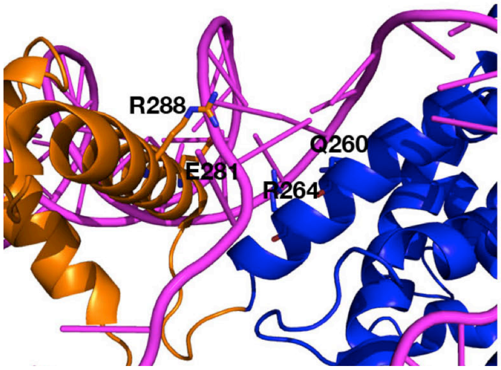

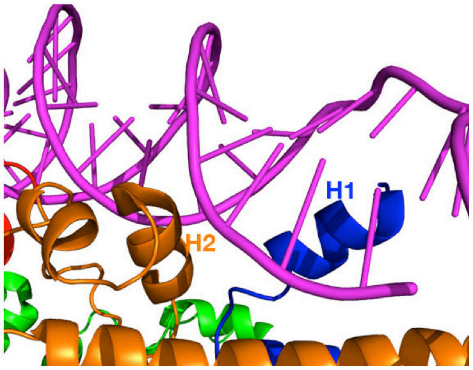

Figure S4, related to Figures 2-3. Comparisons of σ and DNA conformations. (A) In RPc and in *Aae* σ^{54}(ΔRI)-DNA complex (pdb code 5ui5) aligned on their RPoN domains (red and salmon). Color coding as in main figures with *Aae* σ^{54}(ΔRI)-DNA in pale green (CBD), light orange (ELH-HTH), and light magenta (DNA). (**B).** sequence alignment of RI and RIII-ELH-HTH using ClustalW2 (http://www.ebi.ac.uk/Tools/msa/clustalw2/). Residues mutated in Figure 2 are indicated with an *. KLEPN – *K. pneumonia,* ECOLI – *E.coli,* PSEPK – *P. putida,* RHOCA – *R. capsulatus*, SALTS - *S. typhimurium,* AQUAE – *A. aeolicus* **(C)** σ^A region 2 (blue) and region 3 (orange) occupy similar locations relative to promoter DNA with **(D).** σ^{54} RI (blue) and HTH (orange). Residues shown to be important in the *Thermus aquaticus* (Taq) RNAP-σ^{70} (σ^A) RPo structure in interacting with transcription bubble are labeled. The two helices in σ^{54} from RI and RIII-HTH that are positioned similarly to interact with DNA as those in σ^A are labeled.

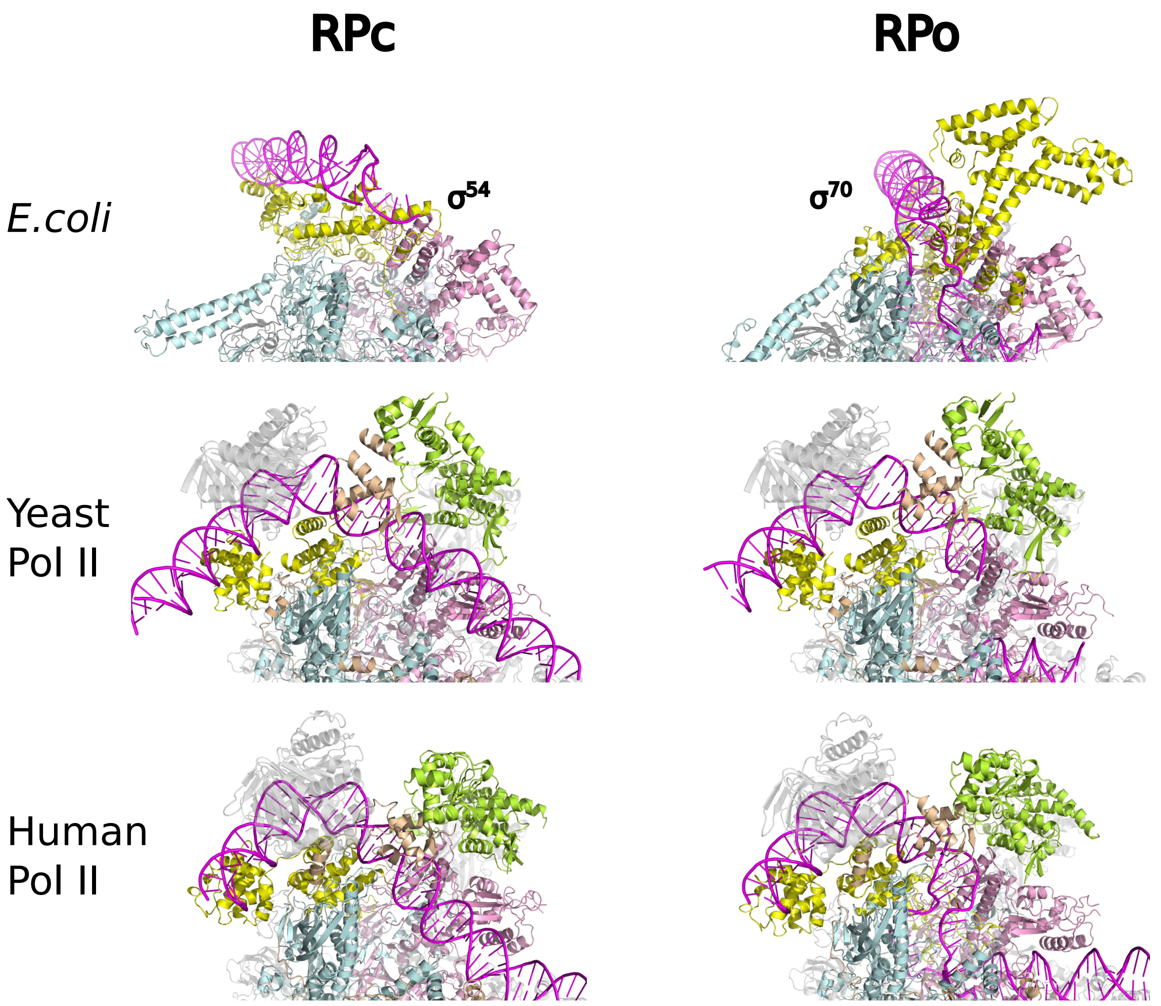

Figure S5, related to Figures 1-3. Comparison of *E. coli* RPc complex (reported here) with those of yeast Pol II (pdbcode 5FZ5), human Pol II (pdbcode 5IYA), *E. coli* open/initiation complex (pdbcode 4YLN) with those of yeast Pol II (5FYW) and human Pol II (5IYB). DNA is shown in magenta. σ and TFIIB in yellow, TFIIF in light orange, TFIIE in green while TBP in grey. The structrues are aligned on the bridge helices**.**

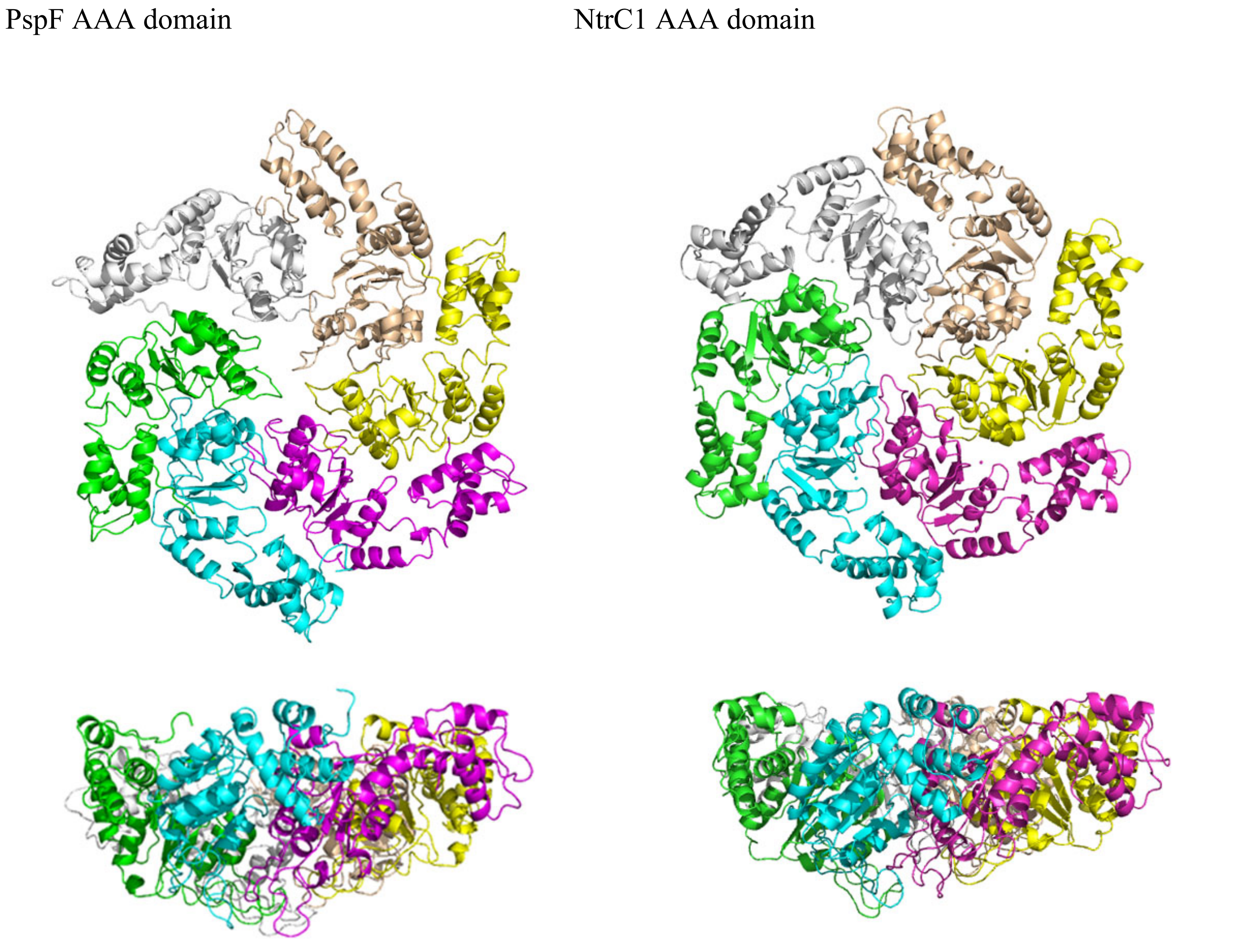

Figure S6, related to Figures 4, 5. Comparison of PspF in RPi with NtrC1 crystal structure (pdb code 4LZZ) viewed from the top (top row) and the side (bottom row) showing the asymmetric arrangement of the hexameric ring in both PspF and NtrC1.
