# Supplementary figures and images for: Structures of RNA Polymerase Closed and Intermediate Complexes Reveal Mechanisms of DNA Opening and Transcription Initiation
Source: Mol Cell. 2017 Jul 6;67(1):106–116.e4. doi: 10.1016/j.molcel.2017.05.010 (PMC5505868; doi:10.1016/j.molcel.2017.05.010)

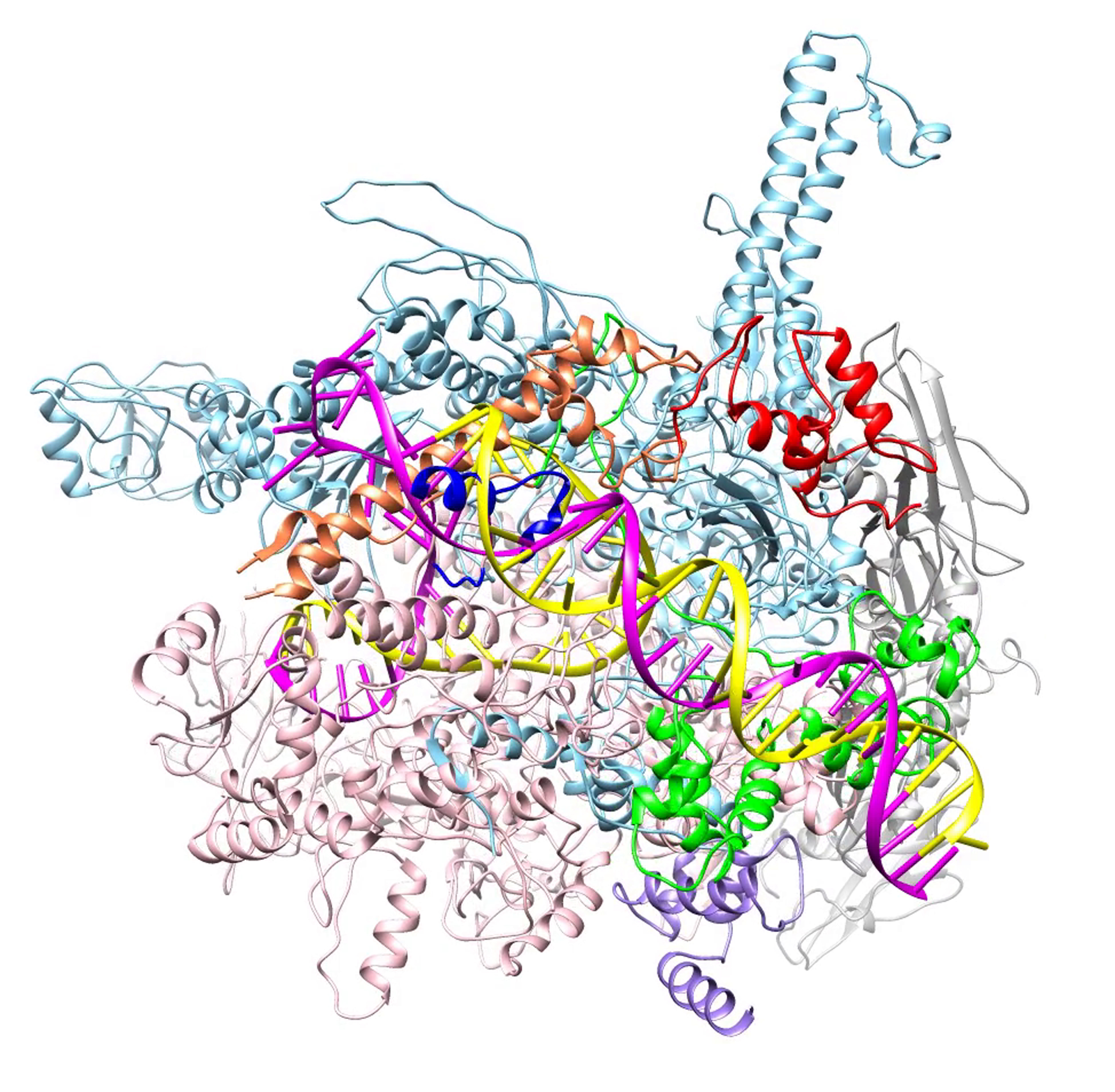

Supplement: Movie S1. Morphing between RPc and RPi Structures, Related to Figure 5 — For clarity, only the structures of RNAP-σ54 are shown. Viewed from the top showing the σ54 ELH and β’-clamp relocations. cyan- β, light pink – β’, blue-σ54 RI, orange – ELH-HTH, green – CBD, red – RpoN. For reference, a transcription bubble (magenta/yellow) from 4YLN, by aligning bridge helices, was also displayed. [file mmc2.jpg]

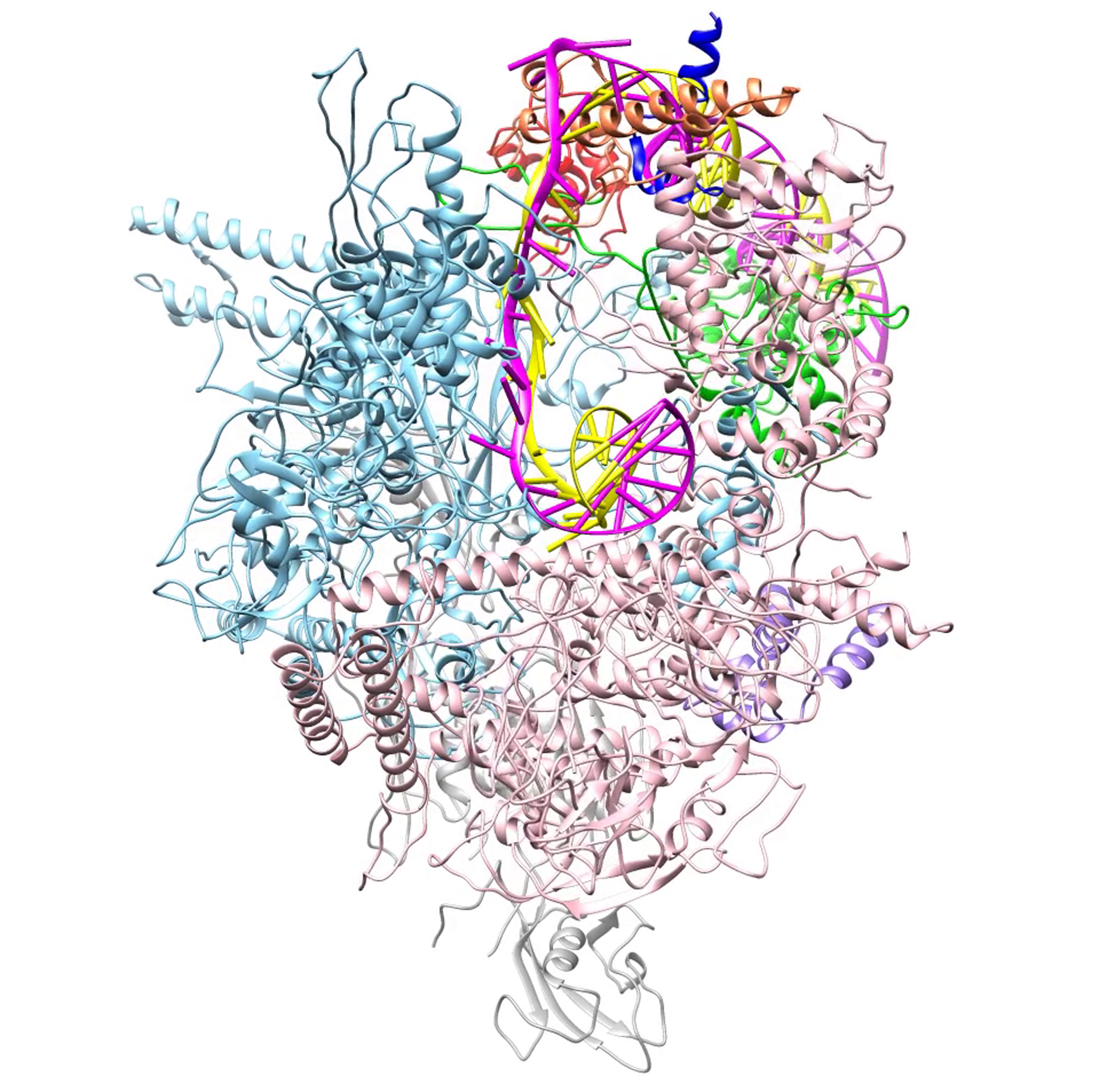

Supplement: Movie S2. Same as Movie S1, Related to Figure 5 — Viewed into the downstream DNA channel showing the cleft opening. [file mmc3.jpg]

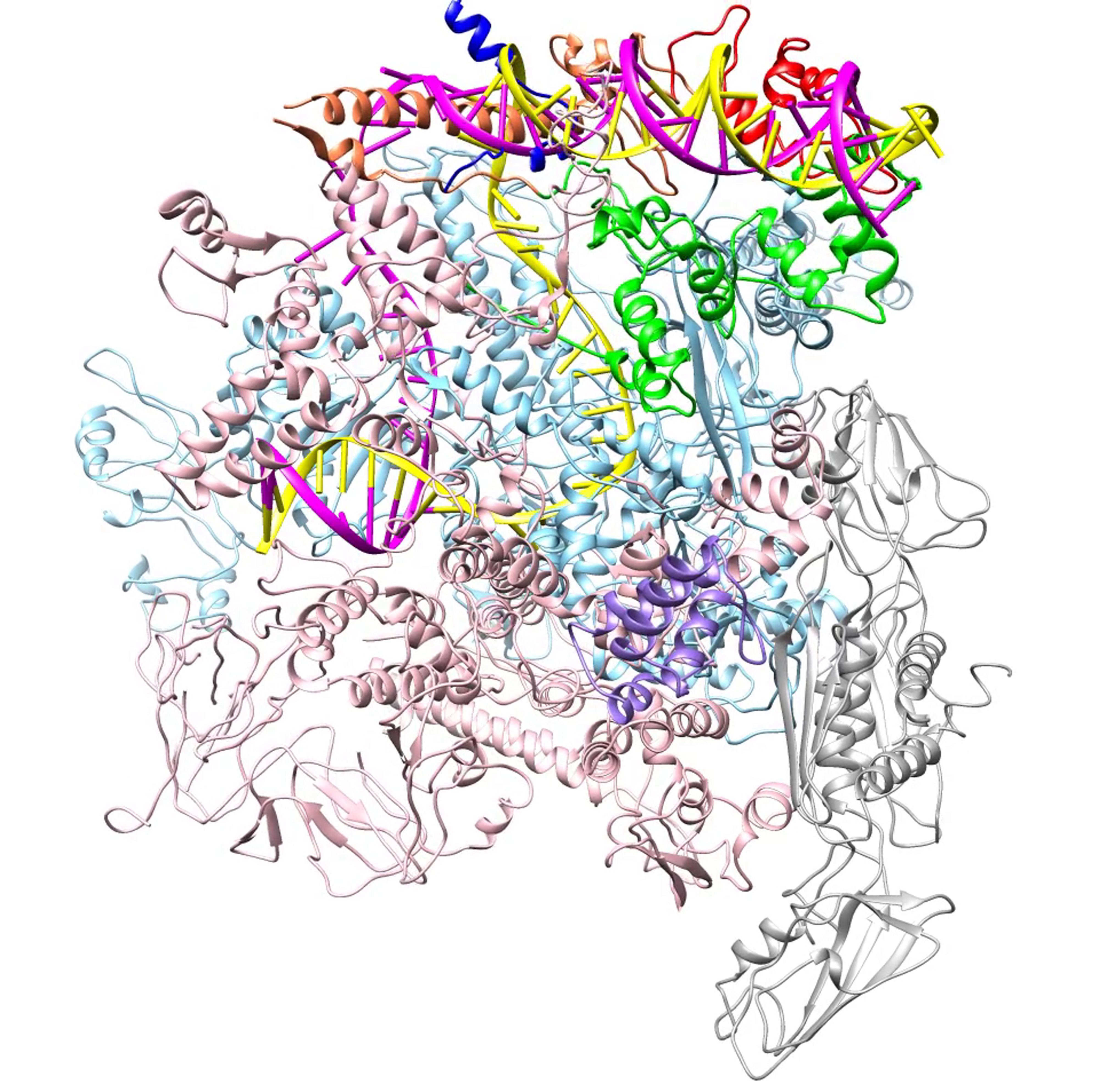

Supplement: Movie S3. Same as Movies S1 and S2, Related to Figure 5 — Viewed from the β’ side showing the relocation of CBD. [file mmc4.jpg]
